# A case study of a bispecific antibody manufacturability assessment and optimization during discovery stage and its implications

**DOI:** 10.1093/abt/tbae013

**Published:** 2024-05-29

**Authors:** Shuang Wang, Weijie Zhang, Baotian Yang, Xudong Zhang, Jing Fang, Haopeng Rui, Zhijian Chen, Jijie Gu, Zhiqiang Chen, Jianqing Xu

**Affiliations:** Biologics Innovation Discovery, WuXi Biologics, 1951 Huifeng West Road, Fengxian District, Shanghai, 201400, China; Biologics Innovation Discovery, WuXi Biologics, 1951 Huifeng West Road, Fengxian District, Shanghai, 201400, China; Biologics Innovation Discovery, WuXi Biologics, 1951 Huifeng West Road, Fengxian District, Shanghai, 201400, China; Downstream Process Development (DSPD), WuXi Biologics, 288 Fute Zhong Road, Waigaoqiao Free Trade Zone, Shanghai, 200131, China; Biologics Innovation Discovery, WuXi Biologics, 1951 Huifeng West Road, Fengxian District, Shanghai, 201400, China; D3 Bio (Wuxi) Co., Ltd., 1101, 11/F, Building 1, No.6, Lane 38, Yuanshen Road, Pudong, Shanghai, 200120, China; D3 Bio (Wuxi) Co., Ltd., 1101, 11/F, Building 1, No.6, Lane 38, Yuanshen Road, Pudong, Shanghai, 200120, China; Biologics Innovation Discovery, WuXi Biologics, 1951 Huifeng West Road, Fengxian District, Shanghai, 201400, China; D3 Bio (Wuxi) Co., Ltd., 1101, 11/F, Building 1, No.6, Lane 38, Yuanshen Road, Pudong, Shanghai, 200120, China; Biologics Innovation Discovery, WuXi Biologics, 1951 Huifeng West Road, Fengxian District, Shanghai, 201400, China

**Keywords:** bsAb, manufacturability, agitation-induced aggregation, protein engineering, developability

## Abstract

The manufacturability assessment and optimization of bispecific antibodies (bsAbs) during the discovery stage are crucial for the success of the drug development process, impacting the speed and cost of advancing such therapeutics to the Investigational New Drug (IND) stage and ultimately to the market. The complexity of bsAbs creates challenges in employing effective evaluation methods to detect developability risks in early discovery stage, and poses difficulties in identifying the root causes and implementing subsequent engineering solutions. This study presents a case of engineering a bsAb that displayed a normal solution appearance during the discovery phase but underwent significant precipitation when subjected to agitation stress during 15 L Chemistry, Manufacturing, and Control (CMC) production Leveraging analytical tools, structural analysis, *in silico* prediction, and wet-lab validations, the key molecular origins responsible for the observed precipitation were identified and addressed. Sequence engineering to reduce protein surface hydrophobicity and enhance conformational stability proved effective in resolving agitation-induced aggregation. The refined bsAb sequences enabled successful mass production in CMC department. The findings of this case study contribute to the understanding of the fundamental mechanism of agitation-induced aggregation and offer a potential protein engineering procedure for addressing similar issues in bsAb. Furthermore, this case study emphasizes the significance of a close partnership between Discovery and CMC teams. Integrating CMC’s rigorous evaluation methods with Discovery’s engineering capability can facilitate a streamlined development process for bsAb molecules.

## Teaser

Statement of Significance: This article presents a case study addressing agitation-induced aggregation of a bispecific antibody via protein engineering. Detailed process including root cause identification, rational design strategies and wet-lab validation is introduced. This study contributes the understanding of aggregation mechanism and offers insights into optimizing bispecific antibody stability for improved downstream applications.

## Introduction

Bispecific antibodies (bsAbs) have gained significant attention in the biopharmaceutical field due to their capability to target two different targets or two epitopes on one antigen, potentially enabling the design of novel therapeutic mechanisms of action (MoA) and enhancing efficacy [1]. BsAbs achieve their specific functions due to their unique molecular structure. However, this necessitates meticulous design and development to guarantee both their efficacy and manufacturability [2, 3].

Manufacturability refers to the characteristics and considerations related to large-scale production and storage. It includes optimizing expression systems, cell line development, upstream and downstream processing, formulation, stability, quality control, scalability, and cost-effectiveness. By addressing these factors, efficient and cost-effective manufacturing processes can be developed to produce high-quality BsAbs products. As an important part of developability, [4] which refers to the likelihood that an antibody candidate will evolve into a manufacturable, stable, safe and effective drug, manufacturability should be evaluated and assessed early in the discovery phase. Ideally, potential liabilities should be identified and mitigated as early as possible [5, 6]. Antibodies with poor manufacturability will bring enormous challenges to Chemistry, Manufacturing, and Control (CMC) development, manufacturing, formulation, storage, transportation and administration, and may even lead to the failure in clinical trial [7, 8].

As one of the most common manufacturability issues, antibody aggregation is highly undesirable. It may complicate the production process, impair biological activity, and increase the risk of immunogenicity [9–12]. Antibody aggregation could be mitigated through diverse approaches, including the development of purification process [13, 14], and formulation optimization [15–17]. However, significant antibody aggregation that leads to product precipitation during downstream production will likely require sequence engineering [10, 18, 19]. The effectiveness of sequence engineering heavily relies on the fundamental understanding of aggregation mechanism at molecular level, mainly involving colloidal stability and conformational stability [20, 21]. Abnormal charge [22] or hydrophobic patches [23] on the antibody surface may induce low colloidal stability, while incompatible residues types in the sequence that are inconsistent with the highly conserved antibody structure might affect conformational stability [24]. Structural insights derived from computational modeling can help elucidate the root causes, and guide the sequence optimization through mutations to enhance either or both stabilities [10, 22, 23]. When different antibodies with aggregation propensities are converted into building blocks and assembled into complex bsAb molecule, or fused into certain bsAb formats, the risks of aggregation could be further amplified [18, 25]. Similar rational design strategies that optimize the colloidal or conformational stability of internal building units, such as single-chain Fv (scFv), are consistently employed to mitigate aggregation of bsAb [18]. Although much of the literature on bsAb aggregation focuses on exploring purification methods to remove the impurities resulting from the innovative format, and less on discussing the underlying physics, the fundamental principles involved are likely consistent.

The engineering efforts to address agitation-induced aggregates, particularly those leading to large visible particles and precipitation, have been reported relatively less frequently [26]. This is likely because such stress assessments are mainly conducted in the CMC stage with materials from large-scale production, [27] while discovery stage prioritizes high-throughput assessment methods for screening a large number of candidates using a limited amount of protein materials [28]. It remains unclear whether the aforementioned underlying molecular-level mechanisms, the colloidal and conformational stabilities, are applicable in explaining agitation-induced aggregation in this context. Furthermore, it is uncertain whether these principles can guide the rational design of engineering solutions, particularly in the complex realm of bsAbs.

Here, we present a case study in which a bsAb, exhibiting a high final yield (206 mg/L, 40 ml transient expression in Expi293) and normal solution appearance during the discovery stage, unexpectedly experienced significant precipitation under agitation stress during 15 L CMC production in CHO-K1. The addition of surfactant, contrary to expectations, failed to resolve this issue. We conducted comprehensive developability assessments and optimization for this molecule, illuminating the fundamental root causes of agitation-induced aggregation and providing the engineering insights to overcome such challenges. Furthermore, our study emphasizes the importance of leveraging experience from CMC perspective and implementing more stringent developability assessment in the discovery stage for complex bsAbs. Early identification and elimination of potential manufacturing challenges in the discovery stage can significantly improve the efficiency of the subsequent development process.

## Materials and methods

### BsAb construction and production

For the construction of X_1_, X_2_, or Y_1_ monoclonal antibodies (mAbs), polynucleotide sequences encoding the heavy chain or light chain of the antibody were inserted into the multiple cloning site (MCS) region of modified pCDNA3.4, with a human antibody heavy chain or light chain signal peptide at the N-terminus of the sequences, respectively.

For the construction of X_1_-scFv and X_2_-scFv, polynucleotide sequences encoding the scFv form (VH-(G_4_S)_4_-VL) of antibodies X_1_ or X_2_ and hIgG1 Fc were inserted into the MCS region of modified pCDNA3.4, with a human antibody heavy chain signal peptide at the N-terminus of the sequence.

For the construction of bsAbs, polynucleotide sequences encoding the scFv form (VH-(G_4_S)_4_-VL) of antibodies X_1_ or X_2_ were fused to the N terminus of the antibody Y_1_ heavy chain with a (G4S)_2_ linker.

Mutations on X_1_ or Y_1_ were introduced by PCR on the basis of wild type sequences, respectively.

The light chain and heavy chain plasmids were co-transfected with a 2:1 ratio into Expi293 cells (Thermo Fisher, A14635). The transfected cells were then incubated at 37 °C, 8% CO_2_, rotating at 120 rpm in shaker for 5 days. On Day 5, supernatant from the Expi293 cells were collected and filtered by a 0.22 μM filter. A Protein A column (GE Healthcare, Cat. 175438) was used for antibody purification. The concentration of the purified antibodies was determined by measuring absorbance at 280 nm. Antibody molecular weight and purity were characterized by sodium dodecyl-sulfate polyacrylamide gel electrophoresis (SDS-PAGE) and size exclusion chromatography high performance liquid chromatography (SEC-HPLC), respectively.

### Differential scanning fluorimetry

Differential scanning flourimetry (DSF) experiments were carried out using a Quant Studio 7 Flex Real-Time PCR instrument (Applied Biosystems). Antibodies were mixed with SYPRO orange dye (Invitrogen cat#S6651) and transferred to a 96-well plate. The plate was then placed in the Quant Studio® 7 Flex Real-Time PCR system, and the temperature was ramped from 26 °C to 95 °C at a heating rate of 0.9 °C/min. The first temperature of protein unfolding transitions was recorded as T_m_1. The value was calculated according to the melt curve using QuantStudio® Real Time PCR software (v1.3).

### Hydrophobic interaction chromatography

HPLC 1260 Infinity II system (Agilent Technologics™) with TSKgel butyl-NPR column (Tosoh cat#0042168) was used to calculate protein retention time. Each sample was diluted to a concentration of 0.5 mg/ml in phosphate-buffered saline (PBS) and 20 μl diluted sample was injected into the column, with a separated flow rate of 0.5 ml/min for 61 mins. The running buffer was prepared by mixing Buffer A (25 mM sodium phosphate, pH 7.0) and Buffer D (25 mM sodium phosphate, 1.5 M (NH_4_)_2_SO_4_, pH 7.0). The separation was performed from 3 to 53 min using a running buffer gradient (Buffer D from 100% to 0%). The UV absorbance was detected at 280 nm to determine the peak retention. The retention time was calculated by integrating all peak areas from 20 to 40 min using software OpenLab CDS Workstation (v2.6.0.691).

### Modeling

The variable region structure of the antibody was modeled using the homology modeling approach named “Model Antibodies” module in Discovery Studio [29]. The antibody’s light and heavy chain sequences were initially annotated in the Kabat numbering scheme to distinguish framework and complementary determining regions (CDRs), and then queried against an antibody database curated from the protein data bank (PDB). Antibody sequences in the database that are close to the search sequence were ranked based on similarity. To build the initial structural model, high resolution (<2.5 Å) and low B-factor (<50) antibody crystal structures, whose sequences of framework regions best matched that of the query sequences, were selected as structural templates for the light and heavy chains. The structural templates of the CDR loops were obtained in a similar way by matching CDR sequences. Three templates of each CDR loop were aligned with the corresponding regions on the initial model. All components were then assembled into a complete antibody structure using the MODELER tool in Discovery Studio, followed by optimization. Finally, the model with the lowest total energy was chosen as the final model for subsequent structural analyses.

### *In silico* mutagenesis

The Y_1_ light and heavy variable sequences were individually aligned with closely related human germline sequences having > 80% identity, using the “Sequence Analysis” module in Discovery Studio. Based on the alignment, the frequency of each reside type at every framework site was examined. If the original Y_1_ residue type did not exhibit the highest frequency, the new residue type from the germline sequences with the highest frequency were utilized to substitute the original residue in the Fv model of antibody Y_1_.

The structural model was annotated and preprocessed at pH 7.4 using “Prepare Proteins” module in Discovery Studio. Subsequently, *in silico* mutagenesis was performed on the proposed framework positions using the “Protein Design” module. The stability changes resulting from germline mutations were assessed by calculating the difference in folding free energy (ΔΔG_mut_) between the wild type and the mutated model. A threshold of ΔΔG_mut_ < 0 kcal/mol was used to select mutations that could potentially stabilize the protein structure. These positions were then chosen for further wet-lab validation.

### **Spatial aggregation propensity** analysis

The Fv model of antibody X_1_ was annotated and preprocessed at pH 7.4 using “Prepare Proteins” module in Discovery Studio. The spatial aggregation propensity (SAP) analysis [30] was performed on X_1_ model utilizing “Protein Design” module in Discovery Studio to identify hydrophobic patches on the surface. High SAP scores indicate highly exposed hydrophobic regions. The SAP score for each protein atom is calculated as the following equation:


\begin{align*} {SAP}_{atom\ i}&=\sum_{\begin{array}{c} Residues\ with\ at\ least\ \\{} one\ side\ chain\ atom\ \\{} within\ R\ from\ atom\ i\end{array}}\\&\left(\frac{\begin{array}{c} SAA\ of\ \mathrm{side}\ \mathrm{chain}\ \mathrm{atoms}\\{}\ \mathrm{within}\ \mathrm{radius}\ \mathrm{R}\end{array}}{\begin{array}{c}\mathrm{SAA}\ \mathrm{of}\ side\ chain\ atom s\ \\{} of\ fully\ exposed\ residue\end{array}}\times Residue\ Hydrophobicity\right) \end{align*}

where SAA is the solvent accessible surface area, R is the radius. The radius used in this work was 5 Å. The SAA of the fully exposed side-chain is precalculated for each standard amino acid from the central residue of an Ala–X–Ala tripeptide in the fully extended conformation. The residue hydrophobicity scale used here is from Black and Mold [31], where Gly is assigned a value of 0. The SAP score for each residue is obtained as the average of its atomic aggregation scores.

### FACS binding

Cells expressing the target protein of antibody X_1_ or Y_1_ were incubated with various concentrations of X_1_ or Y_1_ at 4 °C for 1 hour. After washing with 1 × PBS/1% bovine serum albumin (BSA), the secondary antibody, PE-labeled goat anti-human IgG was added and incubated with cells at 4 °C in dark for 1 hour. The cells were then washed twice with PBS, and re-suspended in 1 × PBS/1%BSA. Mean fluorescence intensity (MFI) of the cells was measured by a flow cytometer (BD) and analyzed by FlowJo.

### Shaking and turbidity test by absorbance spectroscopy at 350 nm.

After diluting samples to 1.5 mg/ml, 400–500 μl of each sample was shook up and down for 50 times in 1.5 ml tube, then the appearance was checked visually. Turbidity was measured by transferring 200 μl of each sample into a 96-well flat-bottom plate, and the SpectraMax M5e (Molecular Devices) parameters were set to a wavelength of 350 nm and a temperature of 37 °C, and then measure the absorbance. The absorbance of PBS and Formulation buffer (20 mM His, 200 mM Arg-HCl, 70 mM sucrose, 0.01% (w/v) PS80, pH 7.0) was measured as background, and will be deducted when calculating the turbidity of each sample. The turbidity of X_1_Y_1_ is defined as 100% in “precipitation level”. The wavelength of 350 nm was chosen because the sensitivity towards turbidity is high at this wavelength [32].

### Large scale CMC production

BsAb production in CMC was expressed in stably transfected CHO-K1 cells grown in HyClone ActiPro culture medium supplemented with Cell Boost 7a and 7b. The post seed culture expansion the cells were transferred to a 15 L or 50 L bioreactor and cultivated at 36.5 °C. When cell density reached 12 × 10^6^ viable cells/ml (takes ~3 days), the temperature was dropped to 33 °C and the culture was allowed to grow for another 11 days before harvest. During this period, pH of the culture media was kept in the range of 6.8–7.2. Amino acid and sugar supplements were added on Day 3, 5, 7, 9, and 11. Cell culture was harvested using depth filtration followed by downstream purification.

## Results

### Precipitation of bsAb under agitation

**Figure 1:**
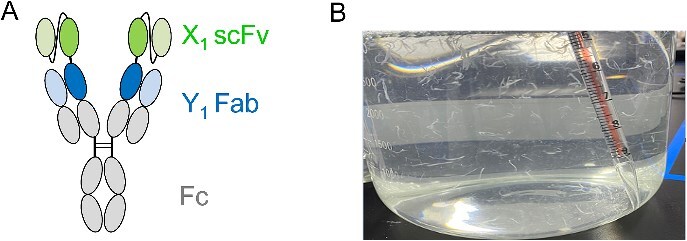
Flocculent precipitation was observed for X_1_Y_1_ during large scale production. (A) Schematic representation of the X_1_Y_1_ bsAb format. (B) Continuous flocculent precipitation observed upon shaking or stirring during large-scale production in CMC.

In this case study, two monoclonal antibodies (mAbs), X_1_ and Y_1_, targeting two different antigens, were utilized to construct IgG1 bsAbs in multiple formats. The molecule X_1_Y_1_ in scFv-mAb format (Fig. 1A), comprising X_1_-scFv linked to the N-terminus of the Y_1_-mAb heavy chain with a (G_4_S)_2_ linker, was chosen as the lead candidate based on its superior functionality (data not shown). Initial assessments were conducted to evaluate the developability of X_1_Y_1_, encompassing transient expression and purification in 40 ml scale, purity and thermostability. After 1-step of purification, the sample achieved final yield to >200 mg/L and purity to > 97% in SEC characterization. DSF showed that the Tm1 of this bsAb is 63.8 °C.

The lead candidate, X_1_Y_1_, was chosen for advancement to a 15 L scale production in the CMC stage. This scale-up production was initiated with the purpose of generating materials for in-vivo study. The 15 L production involved a 14-day upstream cell culture using CHO-K1 cell line, clarification via depth filtration, and a platform downstream process. Unexpectedly, flocculent precipitation was observed in the Protein A affinity chromatography (AC) elution pool, as shown in Fig. 1B. Filtration attempts were made to remove the precipitation but shaking or stirring the solution still led to formation of significant amounts of severe precipitates. Different pH/buffer, excipients and surfactant were evaluated, but shown no effect on the formation of flocculent precipitation. The precipitation can be dissolved in 4 M Urea, suggesting that the precipitation could be proteins. The flocculent precipitation poses significant challenges in downstream processing, including filter clogging, reduced performance, product loss, and stability issues.

### Root-cause identification

Two parental IgG1 antibodies, X_1_ and Y_1_, were individually investigated to explore the root cause of the precipitation issue of the bsAb. Upon shaking, both mAbs exhibited continuous precipitation (Supplementary Fig. S1A), indicating their collective involvement in the bsAb’s precipitation, with X_1_ playing a more significant role. To improve the clarity of descriptions and make comparing samples easier, we assessed the turbidity of all samples and introduced a parameter called “precipitation level”, which was defined as the ratio of turbidity (OD350 value) of the inspected samples compared with that of the bsAb X_1_Y_1_. 10 commercially approved mAbs were produced in-house, formulated in PBS, and their precipitation levels were tested using the turbidity assay. The precipitation levels of these mAbs were found to range from 1% to 5%. It is thus believed that protein therapeutics with a precipitation level <5% could realistically pass CMC assessments with low risk.

hydrophobic interaction chromatography (HIC) and DSF were conducted for the bsAb and parental antibodies to assess their colloidal stability and conformational stability, respectively. The characterized data, along with their precipitation level (after shaking) is presented in Table 1. Parental antibody X_1_ exhibited an unusually long retention time of 39 minutes in HIC, and a normal melting temperature Tm1 of 66.7 °C in DSF, suggesting that the precipitation of X_1_ might be primarily caused by its surface hydrophobicity. In contrast, antibody Y_1_ showed a normal retention time of 28 minutes, but a relatively lower Tm1 of 63.2 °C, suggesting the issues of Y_1_ might result from its lower conformational stability.

**Table 1:** Developability characterizations of bsAb X_1_Y_1_ and its parental antibodies. All samples were analyzed in PBS buffer at a concentration of 1.5 mg/ml. After sample shaking, the precipitation level was defined as the relative turbidity signals of the measured samples compared with that of the wild type bsAb X_1_Y_1_. The OD350 values of turbidity test are listed in Supplementary Table S2.

**Antibody**	**HIC**	**DSF**	**Precipitation level** **(%)**
	**Retention Time** **(min)**	**Tm1** **(°C)**	
X_1_Y_1_	42.3	63.8	100
X_1_	41.0	66.7	114
X_1_-scFv	43.2	70.7	127
Y_1_	29.9	63.2	14

X_1_Y_1_ exhibited an extended retention time similar to X_1_ and a low Tm1 akin to Y_1_, indicating its precipitation might result from the additive effect of two mechanisms. The precipitation level of X_1_ is significantly higher than Y_1_, indicating that it might play a dominant role in the bsAb aggregation. Given that both the IgG and scFv forms of X_1_ showed a similar level of precipitation, its intrinsic surface hydrophobicity, rather than the format change, was hypothesized as the main cause. This positions antibody X_1_ as the primary focus for subsequent engineering efforts.

### X_1_ mutation in X_1_Y_1_ bsAb

**Figure 2:**
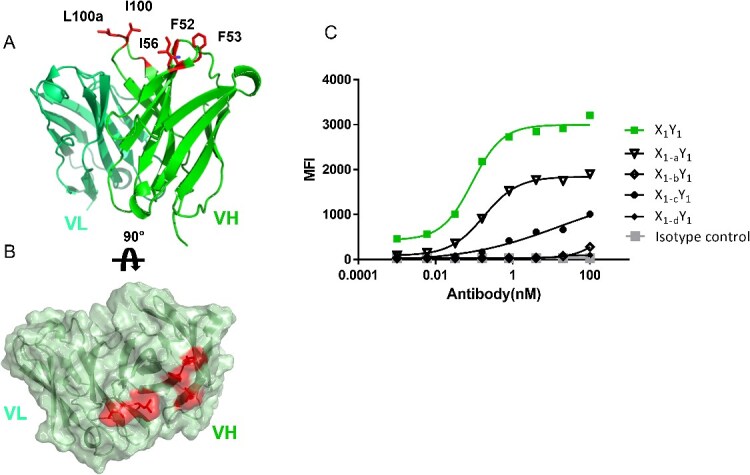
Fv homology model of X_1_, and the hydrophobic patches identified by SAP analysis. (A) Cartoon representation of the modeled structure of X_1_ variable region, with VL on the left and VH on the right. The hydrophobic residues at CDR2 and CDR3 of VH are shown as sticks and colored in red. (B) SAP analysis of X_1_ variable region, showing that that the most hydrophobic patch is corresponding to the hydrophobic residues highlighted in (A). (C) FACS Binding results of X_1_ designs in bsAb to X_1_ targeted cells.

To gain a deeper understanding of the surface hydrophobicity of X_1_ at the molecular level, we constructed a homology model of the Fv region and performed SAP [30] analysis on it. Two significant hydrophobic patches were identified (shown in Fig. 2B), which were spatially proximal to each other. Subsequent sequence and structural analysis revealed the presence of highly hydrophobic amino acids in the CDRs of its heavy chain variable region (VH), as illustrated in Fig. 2A. Residue I100, L100a in VH CDR3 and F52, F53, I56 in VH CDR2 were identified as key residues dominating the hydrophobic patches. Substituting these residues with less hydrophobic amino acids might help reduce precipitation.

However, the heavy chain CDRs are recognized as the most critical CDR loops in an antibody. Modifications in these areas carry a significant risk of causing binding or even functional loss. To mitigate such risks, a conservative engineering strategy was employed. We initiated the engineering on CDR2, given its relatively lower risk compared to CDR3. Design_1 and Design_2 aimed to replace F52 and F53 on VH CDR2 with tyrosine (Y) or histidine (H), with the goal of reducing hydrophobicity while minimizing the impact on binding. In the event that these two conservative approaches were insufficient in reducing hydrophobicity, Design_3 and Design_4 respectively incorporated an additional CDR2 mutation (I56S), and two extra CDR3 mutations (I100S and L100aT) based on Design_1, in an attempt to further increase hydrophilicity. Anticipating that resolving the aggregation issue with X1 could directly tackle the precipitation problem in bsAb, we directly carried out the wet-lab mutation experiments on bsAb X_1_Y_1_.

Table 2 lists the characterization data of the four designs, including changes in their binding capability to the X_1_ target. The Tm1 values were consistent for all the designs, while the hydrophobicity by HIC showed a slight reduction. All mutants reduced the precipitation level of the bsAb, suggesting potential correlation between the X_1_ hydrophobicity and the observed precipitation level. Unfortunately, even the most conservative mutation Design_1 (F52Y, F53Y) resulted in a two-fold loss in cell-based target binding. The remaining mutations in Design_2–4 exhibit a greater loss in binding capacity (Fig. 2C), albeit with a somewhat diminished level of flocculation. The detected binding loss suggested that these hydrophobic residues were indeed crucial for X_1_ binding. Given the absence of an antibody–antigen cocrystal structure in the current case, recovering affinity through rational design alone is challenging. Due to the tight project timeline, alternative option was considered.

**Table 2:** Developability characterizations of all the X_1_-based hydrophobicity reduction designs. All residues were labeled in Kabat numbering. All samples were analyzed in PBS buffer at a concentration of 1.5 mg/mL. After sample shaking, the precipitation level was defined as the relative turbidity signals of the measured samples compared to that of the wild type bsAb X_1_Y_1_. The OD350 values of turbidity test are listed in Supplementary Table S2**.**

	**Antibody**	**Mutations on VH_X**_**1**_	**HIC**	**DSF**	**Binding loss (fold)**	**Precipitation level (%)**
			**Retention time** **(min)**	**Tm1 (°C)**		
	X_1_Y_1_	WT	42.3	63.8	1	100
Design_1	X_1_a_Y_1_	VH: F52Y/F53Y	41.3	63.4	~2*	64
Design_2	X_1-b_Y_1_	VH: F52Y/F53H	38.0	63.4	>1000	50
Design_3	X_1-c_Y_1_	VH: F52Y/F53Y/I56S	39.4	63.6	>1000	49
Design_4	X_1-d_Y_1_	VH: F52Y/F53Y/I100S/L100aT	38.8	63.8	>1000	49

^*^~ 2-fold loss in EC50, ~ 40% loss in top MFI (Fig. 2C).

### Replacement of X_1_ with X_2_ in bsAb

A backup mAb X_2_, with comparable binding (Supplementary Fig. S2) and function (data not shown), was employed to substitute X_1_. The HIC and DSF analyses indicated that X_2_ displayed typical properties (Table 3). No precipitation was observed after shaking. The scFv version of X_2_ was examined in parallel. Although X_2_-scFv displayed a Tm1 at 57.5 °C, which is lower than its IgG1 format at 69 °C, surprisingly, this reduced thermostability did not lead to flocculation. The X_2_-scFv sample remained clear even under intensive shaking stress (Supplementary Fig. S1B). The newly constructed bsAb X_2_Y_1_ inherited these characteristics, displaying the same Tm1 as the X_2_-scFv, and notably improved precipitation behavior compared to the original bsAb X_1_Y_1_ (Table 3 and Supplementary Fig. S1C).

**Table 3:** Developability characterizations of mAb X_2_ and Y_1_, X_2_-scFv, and bsAb X_2_Y_1_. All samples were initially analyzed in PBS buffer at a concentration of 1.5 mg/mL. BsAb X_2_Y_1_ in formulation buffer was also tested. After sample shaking, the precipitation level was defined as the relative turbidity signals of the measured samples compared to that of the wild type bsAb X_1_Y_1_. The OD350 values of turbidity test are listed in Supplementary Table S2**.**

**Antibody name**	**HIC**	**DSF**	**Precipitation level (%)**
	**Retention Time (min)**	**Tm1 (°C)**	
X_2_	27.8	69.0	7
X_2_-scFv	30.7	57.5	9
Y_1_	29.9	63.2	14
X_2_Y_1_	32.1	57.0	37
X_2_Y_1_ (in formulation buffer*)	NA	NA	14

^*^Formulation buffer (20 mM His, 200 mM Arg-HCl, 70 mM sucrose, 0.01%(w/v) PS80, pH 7.0).

The replacement of X_1_ by X_2_, although effectively mitigated the precipitation, did not completely solve the problem. Precipitation persisted in the new bsAb X_2_Y_1_ after shaking and was more pronounced than that observed with each individual component. The fusion of X_2_-scFv and Y_1_ magnify the aggregation. Introducing a formulation buffer [33] alleviated the aggregation to some extent. These observations underscore the significance of addressing the stability concerns associated with Y_1_.

### Rational designs to optimize Y_1_ and bsAb

**Figure 3:**
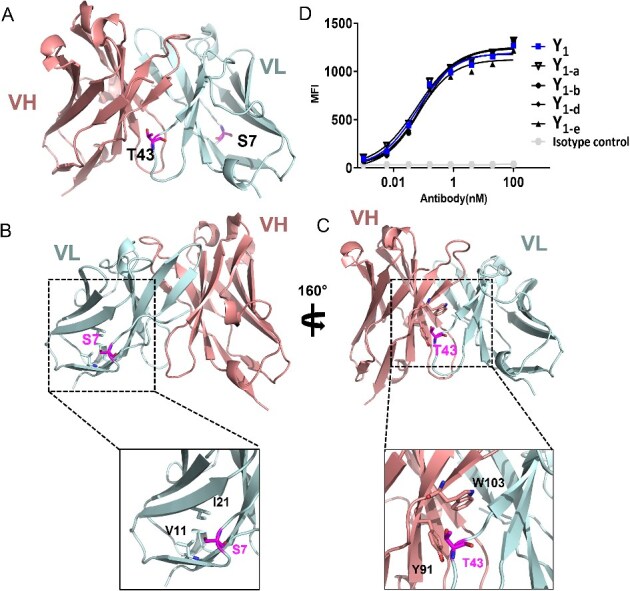
Structural modeling and stability design to optimize mAb Y_1_ precipitation. (A) Cartoon representation of the modeled Fv structure of mAb Y_1_. The positions of S7 and T43 were highlighted in sticks. VL and VH domains were colored in light cyan and salmon, respectively. (B-C) Detailed structural analysis of S7 and T43. (D) FACS Binding of Y_1_ designs to target cells.

The DSF data revealed that the Tm1 of Y_1_ is lower than a typical IgG1 (Table 1), suggesting that the structure of Y_1_ may lack the stability to withstand thermal stress. Such instability might stem from inappropriate residues at specific positions disrupting the local structure. To identify such residues, the variable sequences of Y_1_ were aligned with closely related germlines (> 80% identity) and listed in one panel. The statistics of residue types at each aligned framework position were then calculated. Six positions were identified where the original Y_1_ residue type did not have the highest frequency in the alignment (Supplementary Table S1), suggesting these positions might be potential defects. *In silico* mutations to the residue type with highest frequency in the alignment were performed on the Y_1_ structural model (Fig. 3A) to evaluate stability energy changes (ΔΔG). CDRs were excluded from this analysis to avoid potential binding loss. Four mutants showing energy improvement (Supplementary Table S1) were selected for wet-lab validation. The Tm1 of the entire bsAb X_2_Y_1_ (57.0 °C, Table 3) was determined by the X_2_-scFv component, because X_2_-scFv has lower thermostability than Y_1_ (57.5 °C and 63.2 °C, respectively) and would always unfolds first during DSF characterization, so the Tm1 of X_2_Y_1_ could not reflect the improvement of Y_1_ thermostability. To better track Y_1_ changes in DSF, we implemented these mutations directly on antibody Y_1_.

Table 4 lists the characterization data of the four single mutation designs and one combined mutation design. Most mutants were well expressed except Design_7, which was not further characterized. All the expressed mutants showed no obvious changes in retention time in HIC. Since all the mutations are in the framework region, the antigen binding remained unchanged (Fig. 3D). All the expressed designs reduced Y_1_ aggregation level to some extent. Design_5 (S7P) and Design_6 (T43A), both in the VL region, showed a clear increase in Tm1 of over 2 °C, affirming their success in boosting conformational stability. Design_9, which combined these two mutations, exhibited an additive effect, improving the Tm1 to 68.3 °C, which is close to that of a regular IgG1 antibody [34–36]. Consistently, Design_9 exhibited the lowest precipitation level.

**Table 4:** Developability characterizations of mAb Y_1_ mutants. All residues were labeled in Kabat numbering. All samples were analyzed in PBS buffer at a concentration of 1.5 mg/ml. After sample shaking, the precipitation level was defined as the relative turbidity signals of the measured samples compared to that of the wild type bsAb X_1_Y_1_. The OD350 values of turbidity test are listed in Supplementary Table S2.

	**Antibody**	**Designed mutations on Y**_**1**_	**HIC**	**DSF**	**Binding loss (fold)**	**Precipitation level (%)**
			**Retention time (min)**	**Tm1 (°C)**		
	Y_1_	None	29.9	63.2	1	14
Design_5	Y_1-a_	VL: S7P	30.0	65.5 (+2.3)	<2	9
Design_6	Y_1-b_	VL: T43A	29.7	67.3 (+4.1)	<2	7
Design_7	Y_1-c_	VL: S59P	NA	NA	NA	NA
Design_8	Y_1-d_	VL: N60D	29.8	64.3 (+1.1)	<2	9
Design_9	Y_1-e_	VL: S7P/T43A	30.0	68.3 (+5.1)	<2	4

The analysis of the mutations based on the modeled structure suggested a possible mechanism: S7 is positioned around certain hydrophobic amino acids, such as V11 and I21. The polar residue Serine might disturb the hydrophobic patch and local stability of Y_1_ light chain (Fig. 3B). With the S7P mutation, the hydrophobic potion of Proline might form hydrophobic interaction with neighboring residues. T43, located at the VH-VL interface, is in close proximity to two large side chain residues, Y91 and W103, in VH. T43 might cause clashes in space and disrupt hydrophobic patches, resulting in instability between VH–VL (Fig. 3C). With T43A mutation, the small side chain of alanine could eliminate the steric hindrance. Double mutations further enhanced the stability, alleviating the precipitation level while showing no influence on FACS binding with Y_1_ targeted cells (Fig. 3D).

As previously discussed, many protein properties can cause precipitation, such as insufficient conformational stability or poor colloidal stability. While Y_1_ did not exhibit obvious colloidal stability issues, its lower Tm1 compared with other IgG1 suggests potential conformational stability issues. Subsequent protein engineering based on this hypothesis seems effective. Interestingly, X_2_-scFv, with a low Tm1, did not precipitate noticeably after agitation. One possible explanation is that unfolded X_2_-scFv might have a lower propensity toward aggregation than unfolded Y_1_, possibly due to sequence differences. Another possibility is that DSF and agitation are two different stress-based characterization methods, detecting different aspects of a protein’s conformational stability. Structural defect of Y_1_ might be sensitive to both thermal and mechanical stress, whereas conformational instability of X_2_-scFv might only be sensitive to thermal stress. This difference could stem from the distinct molecular forces maintaining the structural integrity of each protein or from their different unfolding pathways. Further investigation is needed to elucidate the exact underlying mechanism.

The stability-improved mAb Y_1_e_ was subsequently used to substitute the original Y_1_ in the bsAb antibody X_2_Y_1_. Noticeable improvement was observed for the new bsAb, although very slight precipitation can still be seen after shaking the sample in PBS buffer. The issue was completely resolved once the PBS buffer was replaced by a formulation buffer (Table 5, Supplementary Fig. S1C). This data provided a solid support for the transition of X_2_Y_1_e_ to CMC for large-scale production.

**Table 5:** Developability characterizations of the optimized bsAb. Samples were initially analyzed in PBS buffer at a concentration of 1.5 mg/ml. A formulation buffer was tested on the bsAb X_2_Y_1 − e_. After sample shaking, the precipitation level was defined as the relative turbidity signals of the measured samples compared to that of the wild type bsAb X_1_Y_1_. The OD350 values of turbidity test were listed in Supplementary Table S2**.**

**Protein Name**	**HIC**	**DSF**	**Precipitation level (%)**
	**Retention time (min)**	**Tm1 (°C)**	
X_2_Y_1_	32.1	**57.0**	37
X_2_Y_1-e_	33.1	**57.1**	14
X_2_Y_1-e_ (formulation buffer*)	NA	NA	4

^*^Formulation buffer (20 mM His, 200 mM Arg-HCl, 70 mM sucrose, 0.01%(w/v) PS80, pH 7.0).

### Large scale production results in CMC

X_2_Y_1_e_ was moved to CMC for 50 L-scale production. The downstream process flow chart for this 50 L production is detailed in the supporting information (Supplementary Fig. S3). The production concluded successfully, achieving an overall purification yield of 61% and a SEC purity of 98.4% for the drug substance. Notably, there was no occurrence of precipitation throughout the production process. An example of an in-process sample’s appearance is depicted in Supplementary Fig. S4 in the supporting information.

## Conclusions and Discussion

BsAbs show enormous potential in revolutionizing therapeutic approaches across diverse disease areas. However, their complicated physiochemical properties resulting from unnatural molecular formats may bring significant challenges for the development and manufacturing. The focus of this case study is a scFv-based bsAb that experienced significant agitation-induced aggregation. We introduced the detailed problem-solving process, including the root cause identification and sequence optimization via computational and analytic tools. This case study may serve as a valuable reference for the developability evaluation and optimization of bsAbs having similar issues.

Agitation-induced aggregation is believed to result from the adsorption and nucleation of antibodies at the air-water interfaces that are continuously regenerated by mechanic stress [37]. The addition of surfactants is a routine method to mitigate such issues [38, 39]. In our case, the precipitation was so substantial that conventional formulation approaches were ineffective and sequence optimization had to be performed. Engineering interventions targeting surface hydrophobicity and conformational stability proved effective. For example, modifications in hydrophobic residues (Design_1, Table 1) of X_1_ reduced agitation-induced aggregation, revealing a potential correlation between surface hydrophobicity and the visible precipitates induced by mechanic stress. It is unexpected that this intense and prominent surface hydrophobicity did not lead to protein aggregation until the protein was exposed to shaking stress. Our case suggests that prioritizing hydrophobicity reduction and conformational stability improvement is a viable designing engineering strategy for addressing such challenges.

HIC and DSF are often employed as the analytical tools to assess the hydrophobicity and conformational stability of an antibody. Unfavorable HIC or DSF data can provide valuable hint and serve as starting point to investigate the aggregation hypothesis. In this study, the initial evaluation of two parental mAbs using these methods provided crucial insights, leading to the generation of a mechanism hypothesis and the design of a corresponding engineering strategy. In our study, HIC detected significant hydrophobicity on X_1_, which was subsequently confirmed as the primary factor influencing the developability of bsAb. The modifications made to decrease the hydrophobicity of X_1_ led to reduced flocculation level. DSF, a method capable of detecting protein conformational instability under thermal stress, successfully identified the structural defect on Y_1_ that caused the agitation-induced aggregation, and guided subsequent engineering efforts. It’s important to highlight that the low thermostability of X_2_-scFv, as identified by DSF, did not result in aggregation when subjected to agitation stress. Certain molecular interactions maintaining the structural integrity of X_2_-scFv are sensitive to thermal perturbation but somehow insensitive to mechanic perturbation. Our case study confirms the values of HIC and DSF in identifying root causes and offering engineering guidance post-aggregation.

Computational modeling and design have proven to be increasingly useful in addressing antibody developability issues. The current case study further confirmed its advantage in optimizing bsAb. Structural homology modeling, when coupled with the SAP method, identified the amino acids contributing to the high hydrophobicity of X_1_; when coupled with stability energy calculation, identified the key residues influencing the conformational stability of Y_1_. These computational analyses effectively mapped the macroscopic developability issues onto amino acid-level properties, thereby facilitating the design of engineering strategy. Nevertheless, it’s crucial to recognize the limitations of computational methods. For example, a predicted hot spot on Y_1_ did not exhibit expression following germline mutations, as seen in Design_7, Table 4. This underscores the importance of the integration of *in silico* and wet-lab data. Protein designer should devise engineering strategies by leveraging a deep understanding of the molecular basis of antibody developability and the intricate relationship between modeling and experimental data.

Optimizing the developability of antibodies is a complicated and multi-dimensional endeavor. The unique sequence that confers unique functionality to an antibody may also be the source of its suboptimal developability. In this study, the hydrophobicity of X_1_ mainly resulted from a few hydrophobic residues in its CDRs. The engineering strategy was to replace these hydrophobic residues with structurally close hydrophilic residues, intending to achieve both hydrophobicity reduction and affinity maintenance. However, even the most conservative mutation on heavy chain CDR2 was unable to maintain the binding affinity of X_1_. To address the developability challenges arising from CDRs, constructing and screening libraries using mammalian cell display could be beneficial [40], although it was not implemented in the current project due to a tight timeline.

The unnatural format of bsAbs poses greater developability risks and more intricate engineering challenges compared to traditional mAbs. In this study, the bsAb precipitation was attributed to the combined effects of the colloidal instability of X_1_ and the conformational instability of Y_1_. In addition to the challenges inherited from problematic parental antibodies, issues such as intrinsic instability of scFv or unfavorable interactions between parental antibodies could further contribute to the complexity. Occasionally, the assembly of two stable components into a single bsAb molecule could still result in stability loss. For example, despite X_2_-scFv and Y_1-e_ showing good stability individually (Table 3 and Table 4), the straightforward fusion led to increased aggregation (Table 5). These issues are often challenging to predict and address. A higher standard of developability assessment is needed when selecting mAbs as building blocks for constructing bsAbs, as even minor defects in mAbs can be magnified within the complex structure of bsAbs.

With the increased number of bsAb being developed as pivotal immunotherapeutic, there is a growing need for a more comprehensive and rigorous early developability criteria for bsAb. The main purpose of evaluating developability in the early discovery phase is to effectively select good candidates. They are typically performed in a rapid and high-throughput manner while consuming small amounts of materials. Involving CMC scientists with expertise in large-scale manufacturing during the discovery stage can provide valuable insights for developability, especially manufacturability, data interpretation and useful guidance for engineering strategies. As demonstrated in this case, although agitation tests are conventionally conducted in the CMC stage, the issue can be identified and addressed via sequence optimization during the discovery stage, thereby reducing development risks beforehand. Consequently, monitoring precipitation status of bsAb after shaking can be incorporated into the checklist at the discovery stage. Through collaborations efforts between the discovery and CMC departments, more checklists and criteria, particularly tailored for complex proteins like bsAbs, can be established during the discovery stage. When required, early-stage engineering measures can be applied to reduce the likelihood of advancing molecules with developability problems. This cooperative approach aids in streamlining the CMC process, thereby enhancing the successful development of bsAb.

## Supplementary Material

Supplementary_file_for_Review_tbae013

## Data Availability

The data that support this study are openly available.

## Animal Use statements

This is not applicable.
