# Correction to: A case study of a bispecific antibody manufacturability assessment and optimization during discovery stage and its implications

**DOI:** 10.1093/abt/tbae021

**Published:** 2024-08-27

This is a correction to: Shuang Wang, Weijie Zhang, Baotian Yang, Xudong Zhang, Jing Fang, Haopeng Rui, Zhijian Chen, Jijie Gu, Zhiqiang Chen, Jianqing Xu, A case study of a bispecific antibody manufacturability assessment and optimization during discovery stage and its implications, *Antibody Therapeutics*, Volume 7, Issue 3, July 2024, Pages 189–198, https://doi.org/10.1093/abt/tbae013

In the originally published version of the manuscript, the contact details for the corresponding authors were incomplete. These have been emended to read: ``Jianqing Xu: Biologics Innovation Discovery, WuXi Biologics, 1951 Huifeng West Road, Fengxian District, Shanghai 201400, China. Zhiqiang Chen: D3 Bio(Wuxi)Co., Ltd., 1101, 11/F, Building 1, No.6, Lane 38, Yuanshen Road, Pudong, Shanghai, 200120, China.'' instead of: ``Biologics Innovation Discovery, WuXi Biologics, 1951 Huifeng West Road, Fengxian District, Shanghai 201400, China.''

In the section **Author Contributions**, the first paragraph has been altered to read: ``ZC, JX, SW and WZ contributed to the conception and design of the study. SW, WZ, BY, XZ, JF and HR performed the experiments and statistical analysis. JG and ZC contributed to the resource acquisition. SW, JX and ZC wrote the first draft of the manuscript. WZ, BY, XZ and JF wrote sections of the manuscript. JX, ZC, SW and WZ contributed to the manuscript revision. All authors contributed to the revision, read and approved the submitted version.''

A section regarding conflict of interest was erroneously omitted. This has been restored in the article to read:

``**Conflict-of-interest statement**

Shuang Wang, Weijie Zhang, Baotian Yang, Xudong Zhang, Jing Fang, Jijie Gu and Jianqing Xu are current employees of WuXi Biologics and may hold WuXi Biologics' stocks. Haopeng Rui, Zhijian Chen, and Zhiqiang Chen are current employees of D3 Bio and may hold D3 Bio' stocks.''

The emendations have been made to the article.

