# Supplementary material for: A case study of a bispecific antibody manufacturability assessment and optimization during discovery stage and its implications
Source: Antib Ther. 2024 May 29;7(3):189–98. doi: 10.1093/abt/tbae013 (PMC11259756; doi:10.1093/abt/tbae013)
Supplement: Supplementary_file_for_Review_tbae013 [file supplementary_file_for_review_tbae013.docx]

# Supplementary

**Supplementary Table S1: Positions where the residue types of Y_1_ did not dominate (frequency <= 50%) in the alignment with closely related germlines. The residue type frequencies were determined by examining the residue types at each aligned position between Y_1_ and closely related germlines (>80% identity).**

| **Chain** | **Position** | **Y_1_ residue type and its frequency** | **Germline residue type having the highest frequency** | **Mutation** | **Mutation Energy (ΔΔG)** |
| --- | --- | --- | --- | --- | --- |
| VL | 7 | S (0%) | P (78.6%) | S7P | -1.04 |
| VL | 43 | T (0%) | A (100%) | T43A | -1.30 |
| VL | 59 | S (50%) | P (50%) | S59P | -1.30 |
| VL | 60 | N (50%) | D (50%) | N60D | -0.07 |
| VL | 80 | T (0%) | A (100%) | T80A | 0.89 |
| VH | 18 | L (0%) | V (100%) | L18V | 1.05 |

**
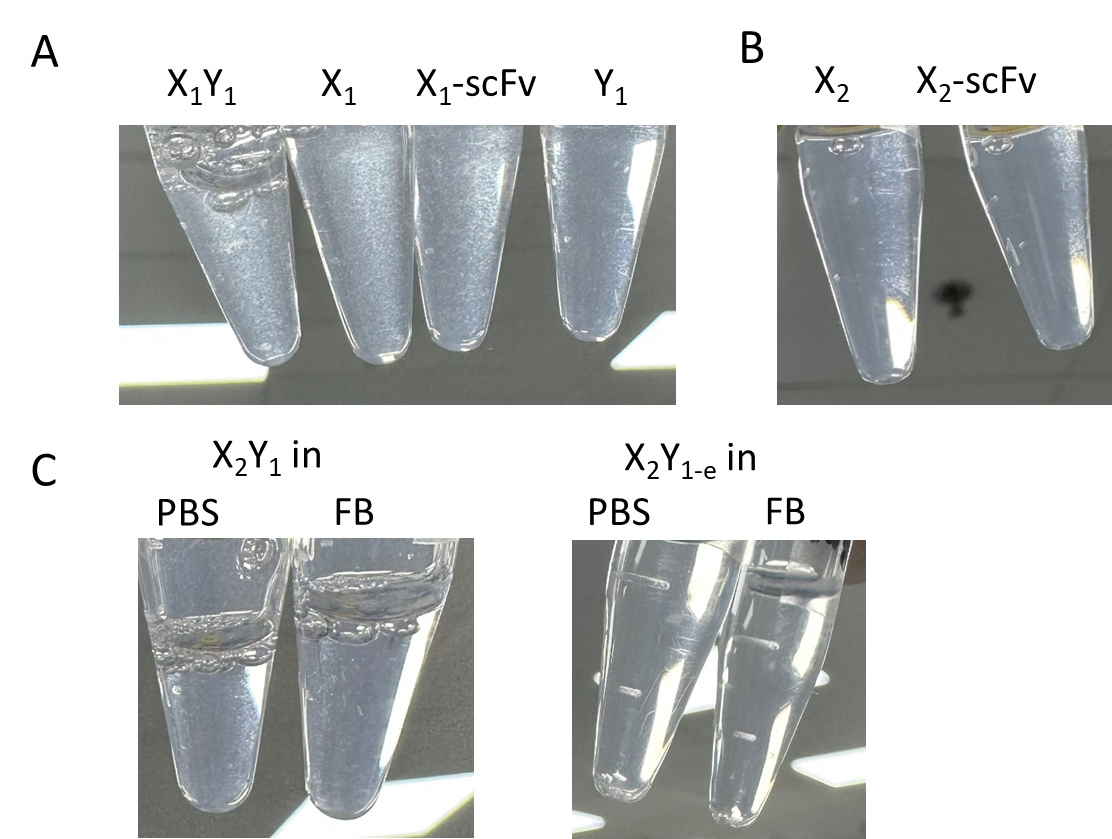
**

**Figure S1: Representative figures of precipitation level of different antibodies.** The images from A to C illustrate the degree of precipitation for the representative antibodies listed in **Tables** 1 through 5, respectively. All samples in A and B were analyzed in PBS buffer. “FB” represents “formulation buffer” (20mM His, 200mM Arg-HCl, 70mM sucrose, 0.01%(w/v) PS80, pH7.0).

**Figure S2. FACS binding results of X_1_ and X_2_ mAbs on target protein expressing cells.**


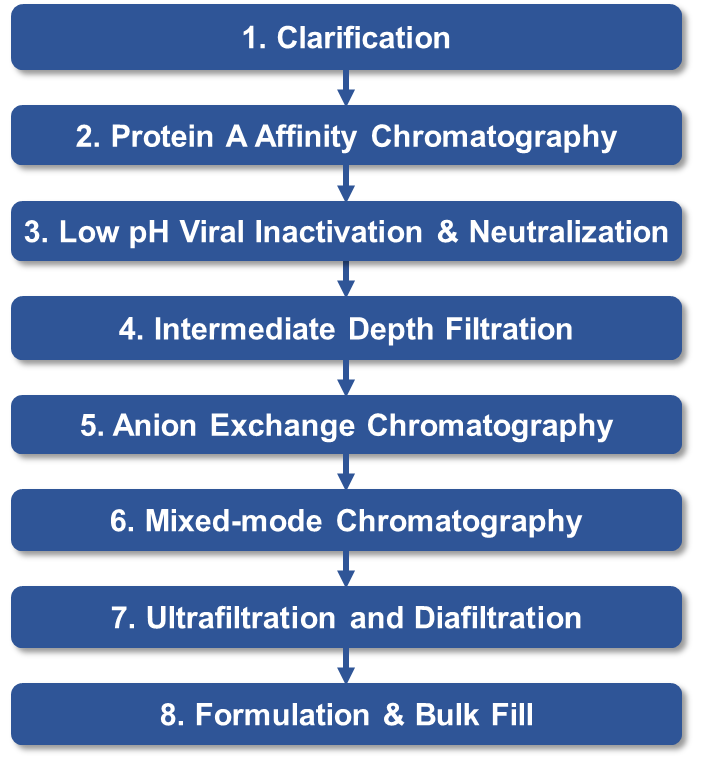


**Figure S3. Downstream Purification flow chart**


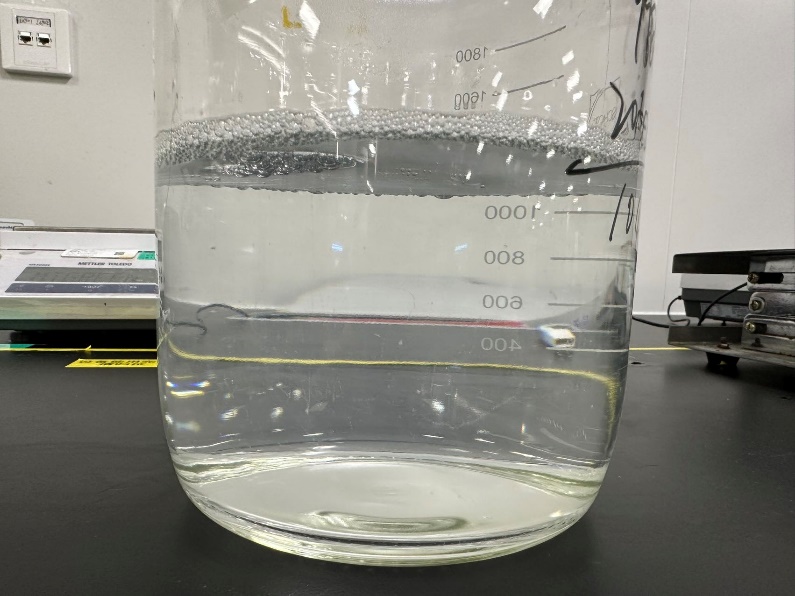


**Figure S4. A representative in-process sample appearance for X_2_Y_1_e_ during CMC production**

**Table S2: Results of turbidity of all samples and the conversion to precipitation level (%).**

|  | Absorbance at 350nm | Precipitation level (%) |
| --- | --- | --- |
| X_1_Y_1_ | 0.2207 | 100 |
| X_1_-scFv | 0.2510 | 114 |
| X_1_-mAb | 0.2797 | 127 |
| Y_1_-mAb | 0.0304 | 14 |
| X_1_a_Y_1_ | 0.1408 | 64 |
| X_1_b_Y_1_ | 0.1114 | 50 |
| X_1_c_Y_1_ | 0.1080 | 49 |
| X_1_d_Y_1_ | 0.1084 | 49 |
| X_2_-mAb | 0.0153 | 7 |
| X_2_-scFv | 0.0202 | 9 |
| X_2_Y_1_ | 0.0811 | 37 |
| X_2_Y_1_ (formulation buffer*) | 0.0320 | 14 |
| Y_1_a_ | 0.0200 | 9 |
| Y_1_b_ | 0.0202 | 9 |
| Y_1_d_ | 0.0176 | 8 |
| Y_1_e_ | 0.0084 | 4 |
| X_2_Y_1_e_ | 0.0312 | 14 |
| X_2_Y_1_e_ (formulation buffer*) | 0.0094 | 4 |

* Formulation buffer (20mM His, 200mM Arg-HCl, 70mM sucrose, 0.01%(w/v) PS80, pH7.0).
